# Trends in Gliosis in Obesity, and the Role of Antioxidants as a Therapeutic Alternative

**DOI:** 10.3390/antiox11101972

**Published:** 2022-10-01

**Authors:** Cindy Bandala, Noemi Cárdenas-Rodríguez, Samuel Reyes-Long, José Luis Cortes-Altamirano, David Garciadiego-Cázares, Eleazar Lara-Padilla, Gabriela Ibáñez-Cervantes, Javier Mancilla-Ramírez, Saul Gómez-Manzo, Alfonso Alfaro-Rodríguez

**Affiliations:** 1 Neurociencias Básicas, Instituto Nacional de Rehabilitación Luis Guillermo Ibarra Ibarra, SSa, Mexico City 14389, Mexico; 2 Escuela Superior de Medicina, Instituto Politécnico Nacional, Mexico City 11340, Mexico; 3 Laboratorio de Neurociencias, Instituto Nacional de Pediatria, SSa, Mexico City 04530, Mexico; 4 Departamento de Investigación, Universidad del Valle de Ecatepec, Ecatepec 55210, Mexico; 5 Unidad de Ingeniería de Tejidos, Terapia Celular y Medicina Regenerativa, Instituto Nacional de Rehabiltación Luis Guillermo Ibarra Ibarra, SSa, Mexico City 14389, Mexico; 6 Hospital Juárez de México, SSa, Mexico City 07760, Mexico; 7 Hospital de la Mujer, SSa, Mexico City 11340, Mexico; 8 Laboratorio de Bioquímica Genética, Instituto Nacional de Pediatría, SSa, Mexico City 04530, Mexico

**Keywords:** gliosis, neuroinflammation, antioxidants, free radicals, obesity, comorbidities

## Abstract

Obesity remains a global health problem. Chronic low-grade inflammation in this pathology has been related to comorbidities such as cognitive alterations that, in the long term, can lead to neurodegenerative diseases. Neuroinflammation or gliosis in patients with obesity and type 2 diabetes mellitus has been related to the effect of adipokines, high lipid levels and glucose, which increase the production of free radicals. Cerebral gliosis can be a risk factor for developing neurodegenerative diseases, and antioxidants could be an alternative for the prevention and treatment of neural comorbidities in obese patients. Aim: Identify the immunological and oxidative stress mechanisms that produce gliosis in patients with obesity and propose antioxidants as an alternative to reducing neuroinflammation. Method: Advanced searches were performed in scientific databases: PubMed, ProQuest, EBSCO, and the Science Citation index for research on the physiopathology of gliosis in obese patients and for the possible role of antioxidants in its management. Conclusion: Patients with obesity can develop neuroinflammation, conditioned by various adipokines, excess lipids and glucose, which results in an increase in free radicals that must be neutralized with antioxidants to reduce gliosis and the risk of long-term neurodegeneration.

## 1. Introduction

Obesity is a global public health problem, linked to a number of comorbidities [1]. One that is rarely clinically addressed in these patients is cerebral gliosis, caused by neuroinflammation, which is activated by molecules such as adipokines as well as lipids and glucose from the diet [2,3]. At the brain level, the hypothalamus acts as a regulator of food intake and energy homeostasis. The integrity of the brain nuclei that regulate appetite-satiety is altered by neuroinflammation, as well as other processes, such as memory and cognition [4]. The prevention, diagnosis and treatment of gliosis must be addressed in patients with obesity, and especially in those who have metabolic comorbidities, points included in this review. Gliosis, if left untreated, can increase the risk of long-term neurodegeneration [5]. The pathophysiology of gliosis includes the increase in free radicals, which perpetuate neuroinflammation [6]. In addition to neurons, the brain is formed by glia, which is further divided into microglia, oligodendrocytes and astrocytes. Microglia represents the brain immune system. Oligodendrocytes produce the myelin that envelops axons to enable efficient neuronal communication. Astrocytes facilitate contact between blood vessels and synapses, detecting neuronal activity and releasing gliotransmitters to modulate brain activity [7,8]. As a physiological adaptive response to damage caused by inflammatory processes in the Central Nervous System (CNS), the brain develops gliosis as a protective measure. One of the factors that produce gliosis is a diet that is high in fat and sugar being sustained for several months [9]. In inflammatory processes of the CNS, it has been reported that astrocytes and microglia are mainly involved in the modulation of metabolic signals in the pro-opiomelanocortin (POMC) region in the mediobasal hypothalamus through their ability to detect glucose and fatty acids. Hypothalamic microglia is activated and undergoes functional and morphological changes when flooded with dietary fat [10,11]. The excessive stimulation of these support cells can lead to gliosis or glial activation and then induce oxidative stress and local inflammation in the hypothalamus, which are causally related to the development of obesity [9,12]. The objective of the current review is to shed light on gliosis as an important comorbidity in obese patients that must be prevented and diagnosed. According to gliosis’ physiopathology, we compiled the most promising antioxidants as novel alternatives to treat this condition.

## 2. Gliosis and Obesity

As a physiological adaptive and protective response to the damage caused by an inflammatory process to the CNS, the brain develops gliosis. This is generated by obesity and mainly occurs in the hypothalamus, causing neuronal loss in the POMC region in the medio-basal hypothalamus, which is widely related to the infundibular nucleus or hypothalamic arcuate nucleus, responsible for weight-related functions and energy homeostasis. Neurons responsible for energy balance, such as POMC neurons, agouti-related protein (AgRP), and neuropeptide Y, are located within these brain structures [13]. Gliosis of the medio-basal hypothalamus has also been linked to comorbid obesity and hypogonadism in men [13,14,15]. One of the factors that produces gliosis is a diet that is high in fat and sugar being sustained for several months. This process interrupts the function of different areas of the brain related to the control of appetite and satiety, causing short circuits of information that prevent the proper control of intake and energy balance [16]. Specifically, in obese patients, this hypothalamic disruption negatively affects weight loss capacity, causing a feedback cycle where the cause turns into the effect. Circulating leptin, the main afferent energy storage signal, decreases its uptake in the hypothalamus due to the gliosis present in this area [17]. Several authors mention that this pathway can also fail because of the stress on different cellular organelles, such as the endoplasmic reticulum [18], among other causes. At present, two clinical trials are in progress, where the relation between gliosis and obesity is being evaluated. In the first study, the authors mentioned that obesity causes gliosis within hypothalamic regions, regulating energy balance and glucose homeostasis. Looking at gliosis by means of an interventional, non-randomized and parallel assignment study, three objectives are being evaluated: (1) the role of Roux-en-Y gastric bypass or sleeve gastrectomy (RYGB) and behavioral weight loss programs in the reversion of hypothalamic gliosis; (2) the extent of gliosis as a successful predictor of weight loss; (3) the timecourse of improvements in gliosis after RYGB and the relationship between these improvements and the short- and long-term efficacy of RYGB over a timeframe of 6 and 12 months [19]. In the second study, the impact of hypothalamic gliosis on appetite regulation and obesity risk in children is evaluated through an observational, cohort, prospective study, where the hypothalamic gliosis is measured by T2 relaxation time, using magnetic resonance, dietary intake in children and bodyweight over a timeframe of two years [20].

## 3. Factors Related to Gliosis in Prenatal and Postnatal Stages

Recent research has shown that inflammation can occur in important areas of the brain, related to the control of intake, energy expenditure and in utero adiposity; factors such as gestational diabetes mellitus and nicotine consumption during pregnancy have been directly linked to neuroinflammation from the primary cerebral vesicles that form different regions of the CNS, related to obesity [21,22]. Vuong et al. demonstrated in animal models of gestational diabetes mellitus (a diet high in fat and sucrose), that newborns presented chronic neuroinflammation, mainly in the pyramidal layer and in the hippocampus, decreasing synaptic integrity, and corroborating the cerebral lipotoxic and glucotoxic effect, resulting in the activation of microglia cells. The study postulates that this condition generates an alteration in the development of the CNS mediated by fractalin receptor (CX3CR1), which is essential to the development of synapses and synaptic plasticity in the hippocampus and cognitive functions [21]. Therefore, the CX3CR1 receptor is an important parameter that should be considered in people who had risk factors in utero, to avoid or to limit brain neuroinflammation, which can predispose the individual to cognitive patterns and uncontrolled metabolic homeostasis, such as resistance to leptin and insulin, resulting in the development of both hedonic and metabolic obesity from an early age. Younes-Rapozo et al. showed that pregnant animals exposed to nicotine and its products during lactation presented microglia infiltration, mainly in the lateral and medial arquate nucleus, the paraventricular nucleus and the lateral hypothalamus, and marked astrogliosis in the paraventricular nucleus [22]. In addition, if these adverse events occur when the brain is undergoing rapid cell growth and is highly vulnerable to alterations by external events, known as the “critical period” of the CNS, it can result in a pathology of brain development. During gestation, adaptive strategies are put in place to deal with the changes that occur during the development of the embryo. At the brain level, the plasticity of nerve cells is also activated, which adjusts to the rapid changes that occur during the formation of the different pathways and structures of the brain [23]. There are numerous studies in the literature about what happens in the CNS at an anatomical and morphological level, derived from the effects produced by prenatal malnutrition, related to long-term deficiencies. These deficiencies have been measured by alterations in brain function and chemical composition and include a decrease in the size of nerve cells, as well as myelination and neuronal dendritic growth, manifesting as a delay in brain development [24]. The hypothesis of the thrifty phenotype is related to poor fetal growth, which undoubtedly affects brain development, causing alterations both at the chemical and morphological levels that are manifested in different parameters. In some cases, this trigger increases or reductions in CNS metabolism, which leads to higher susceptibility to type 2 diabetes or metabolic syndrome [25]. This fact is verified in adults with high rates of intra-abdominal adiposity and who are prone to developing metabolic diseases and energy imbalances, and their medical history shows that they usually come from mothers who suffered a high degree of malnutrition during pregnancy [26]. Obesity derived from malnutrition during gestational development has been related to alterations in brain metabolism, mainly in the nuclei of the hypothalamus, related to metabolism and food intake, encircling the prevention of gliosis in pregnant women. These alterations cause inflammatory processes at both the central level and the peripheral level [27,28], which can trigger metabolic diseases in childhood.

## 4. Role of Astrocytes in the Expression of Glial Fibrillary Acidic Protein in the Processes of Injury-Inflammation of the CNS Due to Obesity

Astrocytes are a variety of glial cells that are involved in several brain functions, such as neuronal migration during CNS development, neuronal maturation, and differentiation, as a guide to the growth of axons and dendrites, and as part of the blood–brain barrier (BBB). Due to the several processes in which these nerve cells are involved, they can undergo morphological and functional alterations due to metabolic changes throughout their life. In addition, the astrocytes that are interconnected by gap junctions are related to the hypothalamic control of food intake. Astrocytes possess a structural protein called glial fibrillary acidic protein (GFAP). Various studies have shown that this protein is mainly expressed in pathological states associated with the injuries that occur in the CNS that cause neuronal death. These lesions increase the number of astrocytes in the damaged region, causing astrogliosis, which produces an increase in the expression of GFAP [29,30]. In the hypothalamic area, the inflammation produced by obesity was derived from a high-fat diet associated with weight gain and increased insulin resistance is related to the alteration in glial cells, causing hypothalamic astrogliosis [31]. Due to these changes in the CNS, it has been proposed that astrogliosis presents a hypertrophic phenotype, developed by astrocytes due to these attacks on the CNS, which results in an “adaptation” that produces a regulation of very specific structural proteins, such as GFAP and vimentin [32,33]. Experimental studies where these inhibit the expression of GFAP and vimentin show that neuronal loss is greater after injury, and that this mechanism is possibly related to an increase in the regeneration potential of CNS. In the opposite case, the expression of GFAP is increased as a response to the inflammatory process and glial injury of the hypothalamus [34,35,36]. Joaquim et al. conducted a study in male rats descended from females with food restriction during pregnancy, finding a decrease in the size of adipocytes in the first generation and an increase in the weight of retroperitoneal fat. Their results showed that astrocytes in the periventricular hypothalamus induced an increase in GFAP expression. This trend was maintained in second-generation male rats, combined with weight gain. The authors concluded that there is a phenotypic transgenerational trend toward overweight and obesity in male rats due to malnourished mothers during gestation. Based on the above, we can say that there is an alteration in gene expression that results in an elevated regulation of GFAP and inflammatory mediators, in addition to adipokine receptors, due to overweight/obesity [34,36]. These effects are derived from the defensive response of astrocytes to inflammatory action and injury, which also causes their morphological and functional remodeling, which can cause neuronal hypertrophy [37]. Debarba et al., in studies carried out in rats that at the neonatal level, showed underfeeding as well as overfeeding, which is generally known as neonatal malnutrition, causes morphological changes in the glia in addition to metabolism alterations that lead not only to increased GFAP expression but also increased expression of T cell protein tyrosine phosphatase (TCPTP) and alterations in connexin 30 expression, molecules related to the regulation of proinflammatory cytokines in the hypothalamic astrogliosis. The inflammatory processes associated with neonatal malnutrition develop a metabolic programming that breaks the energy balance. Therefore, both astrocytes and microglia conform with the neuron environment, providing it with a favorable microenvironment to maintain its metabolic functions and synaptic plasticity. When injury or inflammation occurs, astrocytes induce GFAP expression, while microglia favor the expression of allograft inflammatory factor 1 (AIF-1), to counteract the neuronal damage caused by inflammation resulting from malnutrition [38]. Astrocytes, mainly of the fibrous type, and microglia are dynamic cells that are important to maintaining the microenvironment surrounding neurons and regulating neuronal functions, such as synaptic plasticity and metabolism. In response to CNS insults, both cell types rapidly respond and develop a reactive phenotype called reactive gliosis, characterized by the upregulation of specific structural proteins, such as GFAP in astrocytes and AIF-1 in microglia [33,39,40].

## 5. Role of Leptin, Interleukin-6 (IL-6) and Tumor Necrosis Factor-α (TNFα) in Brain Proinflammatory Processes

### 5.1. Leptin

Leptin is a hormone released from adipose tissue in proportion to the level of adiposity. From the circulation, leptin passes through the BBB to the brain [41,42] via signals from its leptin receptor (LepRb), which is widely distributed throughout the whole brain and particularly high within the arcuate nucleus of the hypothalamus (ARH), to attenuate food intake and decrease body weight [43,44]. When the action of leptin and insulin decreases, poor systemic glucose tolerance appears and this deficiency, in turn, has been related to proinflammatory signaling in the hypothalamic area. The combined action of leptin and insulin has led to several pieces of research in biological models. Morabito et al., in an animal model of a high-fat diet (HFD) (60%) for 30 weeks, observed that leptin activity decreased in inflamed areas of the brain; despite the administration of exogenous leptin, these animal’s weights did not decrease. However, caloric restriction caused the animals to reduce their weight and visceral fat, restore leptin and insulin levels, and decrease cerebral gliosis [45]. Inflammation in the hypothalamus and peripheral tissues induces the activation of stress-related kinases, such as c-Jun N-terminal kinase (JNK) and IkB-kinase (IKK), which inhibit mediators of leptin pathways and insulin. This insulin/leptin resistance in critical neurons of the hypothalamus increases the metabolic set point of body weight, promoting a gradual increase in adiposity [46]. It has been described that one of the causes of the infiltration of astrocytes, glia and microglia in the hypothalamus is due to a similar process to that which occurs in visceral adipose tissue, where macrophages also infiltrate. In adipose tissue, the arrival of macrophages and their activation induces the expression of interleukin 6 (IL-6) and tumor necrosis factor-alpha (TNF-α); meanwhile, in the hippocampus, these cytokines activate astrocytes and microglia [47,48,49,50]. In this context, activated microglia cells respond to different neurotoxic stimuli, showing phagocytic functions and the production of proinflammatory mediators such as cytokines, chemokines, reactive oxygen species and nitric oxide with a neuroprotective purpose. However, the prolonged activation of these cells can trigger neurodegenerative processes and even neoplasia; it has even been proposed that leptin also acts as a proinflammatory cytokine [15,46,51,52,53]. Pretz et al. administered a dose of a leptin antagonist PESLAN (100 µg/kg/day) to mice, in order to block its signaling because it was increased by diet-induced obesity. The authors found that leptin signaling was partially reduced, and the opposite effects were exerted, leading to an improvement in glucose metabolism; therefore, they propose that this mechanism is possibly regulated by a decrease in hypothalamic astrogliosis and microgliosis [54]. However, it has been proposed that the insulin effect on the hypothalamus results in the suppression of hepatic glucose output, and that intranasal insulin administration suppresses endogenous glucose output in lean men. However, in overweight or obese insulin-resistant men, the same dose of intranasal insulin is ineffective [55]. Balland et al., in a study in mice, showed that pathological leptin signaling in human retinal astrocytes (HRA) underpins impaired hypothalamic insulin signaling and this explains, in part, the failure of leptin action insulin in the hypothalamus–liver axis, which suppresses hepatic glucose production (HGP), leading to its alteration. This insight into leptin and insulin signaling in HRA neurons led them to hypothesize that hyperleptinemia in obese-induced mice might lead to an imbalance between leptin and insulin signaling in HRA neurons and, consequently, affect the regulation of glucose homeostasis in obesity [56,57]. The mechanism of action that affects glial cells shows that diet plays an important role, so that HFD increases the expression of leptin receptors in astrocytes [58,59], inducing localized astrogliosis between anorexigenic neurons and blood vessels in the arcuate nucleus of the basal medial hypothalamus, altering metabolism and food intake. This hypothalamic inflammation is involved in the process of leptin and insulin resistance, leading to obesity and, quite possibly, diabetes [60]. There are other structures of the brain that can be affected by these neuroinflammation processes, such as the frontal cortex, the parietal cortex, the basal ganglia, the thalamus, the hippocampus, and the cerebellum, which are mainly related to high-level cognitive functions. Based on what was described above, we can say that the increase in leptin levels with a high-fat diet favors an increase in body weight, as well as an increase in hypothalamic astrogliosis and microgliosis, in addition to neuronal damage in the different areas of the hypothalamus [61].

### 5.2. IL-6 and TNF-α

Interleukin 6 (IL-6) is a cytokine that has proinflammatory activity when released as adipokine [62] and, on the contrary, is anti-inflammatory when released as myokine. In obese patients, mainly in visceral and abdominal fat, hypertrophic adipocytes, together with macrophages, release IL-6 as part of the chronic low-grade inflammation that occurs in obese patients [62,63,64]. IL-6, together with TNF-α, are molecules that can trigger pathophysiological processes related to common comorbidities, such as type 2 diabetes [65], systemic arterial hypertension and endothelial damage [66], among others; however, an important comorbidity to consider is neuroinflammation [67]. Circulating proinflammatory cytokines such as IL-6 can cross the BBB and reach the hypothalamus, activating microglia through the signals emitted by endothelial cells and glial cells [68].

The cytokine TNF-α is one of the main mediators in inflammatory diseases [69], it has been shown that nutrition can have an immediate effect on circulating markers of inflammation. Proinflammatory cytokines, including TNF-α, are secreted by white adipose tissue macrophages, and it has been reported that, within a few hours of eating a fatty meal, a transient increase in these proinflammatory cytokines is observed in the circulation [70,71]. In obese patients and rodents, low-grade chronic systemic inflammation is characterized by an increase in circulating inflammatory markers, such as C-reactive protein, TNF-α, interleukin-1 (IL-1) and IL-6 in many metabolic organs, including the pancreas, muscle, and liver, as well as in the brain [72,73]. TNF-α has been implicated in hypothalamic proinflammatory signaling on the development of resistance to anorexigenic hormones, such as insulin and leptin. Alterations in these hormones in the hypothalamus cause an imbalance in feeding and thermogenesis, which leads to increased body weight and obesity. The gene expression of TNF-α has been widely associated with the induction of the hypothalamic inflammatory process [69,74,75,76,77,78,79,80]. TNF-α has been proposed as the link between neuroinflammation and the development of insulin resistance and type 2 diabetes mellitus [81]. In the hypothalamus, both astrocytes and microglia have been reported to be activated by HFD. The hypothalamic regulation of energy balance is rapidly and severely compromised by overnutrition, particularly a diet high in saturated fat, which induces inflammation [72]. Obesity and HFD produce reactive gliosis, which compromises the structure and function of the hypothalamus [80]. Hypothalamic inflammation is proposed to be triggered by the long-chain, saturated-fatty-acid-induced activation of toll-like receptor 4 (TLR4) are present on microglia [77,82]. In this context, cytokines such as TNF-α are produced and act on hypothalamic neurons, resulting in the inhibition of leptin and insulin signaling systems, causing obesity [75,76,77,80,83].

## 6. Oxidative Stress in Relation to Gliosis and Obesity

Free radicals are the product of the oxidation of energy molecules such as sugars and fats. Organs such as the liver, pancreas, adipose tissue and, to a relative extent, the brain, metabolize large amounts of energetic molecules, either for immediate use, storage, the regulation of its serum concentration, or processing. Oxidative stress and inflammation have a close relationship in obese people and obesity animal models; adipose tissues are usually infiltrated by macrophages that respond to inflammatory signals. It is known that, during infection or in the presence of foreign material, macrophages form or generate super oxidant agents for the phagocytosis of microorganisms or cellular debris. Thus, it is very likely that this characteristic is stimulated by diets rich in fats and sugars that could provoke an inflammatory process in the adipose tissue where the macrophages themselves arrive [75]). The same mechanism occurs in brain nervous tissue, where astrocytes or glial cells play a similar role to macrophages [13,82]. Treatment with antioxidants or the removal of molecules that contribute to oxidative stress has been able to reverse the process of gliosis in the hypothalamus of the brain. It is known that, in obese patients and obese rodent models, adipose tissues express high levels of TNF-α [84], and over-express other proinflammatory cytokines such as lipopolysaccharide (LPS), interferon-gamma (IFN-γ), TNF-α, interleukin-1beta (IL-1ß), interleukin-12 (IL-12) and IL-6 associated with insulin resistance that allow for the recruitment of macrophages and other types of leukocytes [85,86]. In this context, it has been thought that neuronal-type cells and glial-type cells such as astrocytes could respond differently to a diet of saturated fatty acids such as palmitic acid (PA). Thus, PA in astrocytic-like cells (T98G) generates greater cytotoxicity and causes a higher production of reactive oxygen species (ROS) compared to the neuronal model of the SH-SY5Y cell line. This intracellular increase in superoxidant agents such as hydrogen peroxide (H_2_O_2_) and extended lipid peroxidation (measured as thiobarbituric acid reactive substances levels) causes cell death by apoptosis in both cell types, and, in turn, apoptosis induces neurotoxicity and glial toxicity [87]. Obesity can then lead to the deterioration of certain tissues, and specifically neural tissue, due to the effects of the inflammatory processes and oxidative stress. One of the enzymes that relates these two processes is NAPDH oxidase (NOX), which is a proinflammatory and prooxidant enzyme [88]. In a study conducted on knock-out mice for the catalytic subunit gp91phox (known as NOX2) of NOX, these mice (NOX2KO) were compared with wild-type mice and the authors observed that when fed diets rich in saturated fat, both types of mice increased their weight in a similar way. However, NOX2-deficient mice have smaller visceral fat deposits compared to wild-type mice, in addition to their attenuated visceral adipocyte hypertrophy and the decreased macrophage infiltration of visceral fat [89]. These NOX2-KO mice also showed improved glucose regulation, and it was determined that the cells that express the NOX2 protein are mainly the macrophages infiltrated in the visceral adipose tissue. Several groups used murine models to determine that diets with highly saturated fats cause brain damage [13,73,90]. NOX2-deficient mice with diets rich in saturated fats do not decrease cerebrovascular integrity markers; they maintain that synaptic density and gliosis markers do not increase. These results indicate that NOX2 can be used as a pathogenic marker that is overexpressed and activated during the high consumption of diets rich in saturated fatty acids and contributes to the formation of superoxidant agents [89]. In another study, it was observed that the GFAP, as a gliosis index, increases the expression of NF-kB, interleukin-8 (IL-8), TNF-α, IL-1β and IL-6, as inflammatory mediators; phospho-ERK (p-ERK), heat shock protein-60 (Hsp60) inducible nitric oxide synthase (iNOS), as neurodegeneration stress-marker proteins; and ROS levels increased as well in the brain of HFD-fed mice. However, the use of glucagon-like peptide-2 (GLP-2) significantly attenuated these changes, indicating that this compound can be used as a neuroprotector, as it improved the neuroinflammation and oxidative stress in HFD animals [91,92]. It has also been shown that HFD promotes cognitive decline through mechanisms as the increased expression of proinflammatory adipokines (TNF-α and IL-6), upregulation of chemotactic adipokines (monocyte chemoattractant protein-1, MCP-1), and an increase in reactive microgliosis and astrocytes. It has been proposed that HFD decreases the brain-derived neurotrophic factor (BDNF) involved in cognitive impairment, probably by oxidative stress and ROS formation. In HFD-fed gerbils, it has been observed that hypertrophied microglia and increased expressions of TNF-α, IL-1β and the mammalian target of rapamycin (mTOR) in the brain probably cause ROS generation [93,94,95]. On the other hand, it has been shown that the HDL levels linked to astrocyte function avert reactive gliosis in hypothalamic astrocytes by improving mitochondrial bioenergetics [95,96]. This opens an important area of knowledge regarding the involvement of oxidizing agents on the development of hypothalamic gliosis, indicating an important role for NOX2, HDL or GLP-2.

## 7. Gliosis in Obese Patients, a Risk Factor for Degenerative Diseases

Gliosis is a non-specific reactive change in glial cells in response to any damage to the CNS, and this response involves the proliferation of several different types of glial cells. Obesity increases peripheral immune cell migration to the CNS and stimulates gliosis, increases sympathetic neuron-associated macrophages, and depletes methyl CpG-binding protein 2 in brown adipose tissue. It increases neuropeptide Y in the CNS, inducing hypothalamic proinflammatory cytokine expression and proinflammatory signaling; thus, the activation of all these mechanisms results in neurodegeneration [97]. Several reports revealed that chronic exposure to a high-fat diet, particularly diets high in saturated fat, contributes to obesity-associated gliosis and plasticity of the astrocytic process. Neurogenic inflammation occurs due to the increased oxidative stress in neurons, promoting mitochondrial and leptin signaling dysfunction in oligodendrocytes, and increasing the gene expression of microglial markers, causing myelin disorders and hypothalamic neuropathy [80,98]. Low- to standard-fat diets reduce microglial activation and reverse hypothalamic inflammation [99]. Diet-induced microgliosis has been recognized in human and laboratory models of neurodegenerative diseases such as Alzheimer’s and Parkinson’s disease and provides a possible mechanistic link between obesity/type 2 diabetes and accelerated memory and cognitive decline [100]. Obese individuals are at greater risk of developing age-related cognitive decline, vascular dementia, mild cognitive impairment, Alzheimer Disease [101], Parkinson’s and Huntington’s disease [102,103]. Kivimäki et al. reported that hazard ratios per 5-kg/m^2^ increase in BMI for dementia were 0.71 (95% confidence interval = 0.66–0.77), 0.94 (0.89–0.99), and 1.16 (1.05–1.27) when BMI was assessed 10 years, 10–20 years, and >20 years before dementia diagnosis [104]. Xu et al. reported that overweight people and obese patients (BMI > 30) developed dementia at midlife at a mean odds ratio of 1.71 and 3.88, respectively [105]. In contrast, the pharmacological inhibition of microglial activation in obese subjects was associated with the prevention of obesity-associated cognitive decline [106].

## 8. Diagnosis of Gliosis

Cerebral gliosis has been qualitatively detected by nuclear magnetic resonance specifically in the T2 channel in brain mapping in vivo. However, Magnetic Resonance Imaging (MRI) turned out to be a valuable diagnostic tool: given that, in the T2 signal, the luminosity is increased, a quantitative measurement is possible using a standardized approach to measurements at hypothalamic levels. Using the longer T2 relaxation time, very reliable results can be obtained to the extent that they can be correlated with the histological findings that have been reported in rodents as well as in humans [107,108]. MRI has become an indispensable test for the evaluation of hypothalamic gliosis in vivo in humans [108,109,110]. MRI has mainly been employed for clinical research purposes, but not in clinical day-to-day practice, since most of the population cannot afford it; in addition, its invasiveness greatly limits its application. This prompts the necessity of other diagnostic options, such as serum markers, which could be useful for the qualitative and quantitative diagnosis of gliosis in people with obesity. To date, only BMI has been shown to be negatively correlated with the size of brain structures such as the hippocampus. [111]. One recent indicator that has been proposed is the evaluation of cognitive impairment, because gliosis has been related to loss of concentration, difficulties in learning and memory loss, and changes in behavior [16,107].

Although postmortem studies are difficult to extrapolate to clinical practice, immunohistochemistry in gliotic human brain tissues obtained from autopsies has demonstrated an increase in the expression of GFAP, Aldehyde Dehydrogenase 1 Family Member L1 (ALDH1L1), Glutamate Aspartate Transporter (GLAST), CX3CR1, integrin alpha M subunit (CD11b), and ionized calcium-binding adapter molecule 1 (IBA1) [40,112]. All the above empathize the need to generate new diagnostic methods for gliosis that could be more accessible and safer for patients with obesity.

## 9. Strategies to Reduce Cerebral Gliosis

Different measures have recently been proposed to minimize the gliosis described in patients with obesity and insulin resistance; one of those is the consumption of prebiotics, probiotics, and symbiotics. These substances can restore the gut–brain axis, improving hippocampal plasticity, neuronal cell mitochondrial function, and slowing or stopping microglia activation [113]. Chunchai et al. showed that an HDF animal model developed several disfunctions, such as intestinal inflammation, peripheral insulin resistance, and CNS alterations such as impaired hippocampal plasticity, an increase in free radicals in different brain regions with mitochondrial dysfunction, hippocampal apoptosis, microglia infiltrates and decreased cognition. The authors showed that, by restoring the gut microbiota using prebiotics, probiotics, or symbiotics, improvements were observed in cognition in obese, insulin-resistant animals. Likewise, a decrease in brain inflammation, oxidative stress and apoptosis of the hippocampus was observed, and mitochondrial dysfunction and alterations in microglia cells were improved [114]. Kang et al. proposed that certain types of physical exercises have neuroprotective effects by reversing gliosis induced by HDF; they showed that animals can reduce proinflammatory cytokines under treadmill exercises (ER) by inhibiting TLR-4, and this is also related to the decrease in the expression of the microglia activation marker (CBAM-1 and fibrillary acidic protein). The authors also showed that the ER increases the antiapoptotic protein Bcl-2 and decreases Bax in the hippocampus compared to sedentary rats [115]. It is worth mentioning that some studies have shown a neuroprotective effect of some compounds found in soy, such as isoflavones, decreasing the apoptosis of neurons due to toxic effects, such as glycoxins and oxidized LDL [116]. However, more studies need to be conducted in this regard.

## 10. Protective Role of Antioxidants in Gliosis

**Figure 1:**
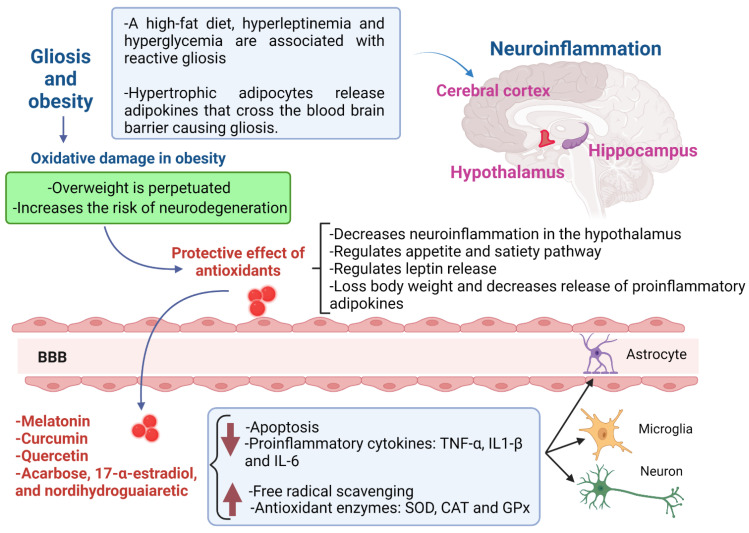
Gliosis in obese patients and the protective role of antioxidants in neuroinflammation. The gliosis is promoted by a high-fat diet, leptin and hyperglycemia in obese patients; this provokes neuroinflammation, mainly in the hypothalamus, which perpetuates overweight, because the satiety-appetite signaling is disrupted. The most promising candidates for the treatment of gliosis in obese patients are melatonin, curcumin, quercetin, acarbose, 17-α-estradiol and nordihydroguaiaretic. Two main antioxidant mechanisms exert neuroprotection: a decrease in apoptosis, leptin release and proinflammatory cytokines, and an increase in free radical scavenging and antioxidant enzymes. The cells that are protected by antioxidants could be the astrocytes, microglia and neurons. BBB, blood–brain barrier; TNFα, tumor necrosis factor alpha; IL-1β, interleukin 1β; IL-6, interleukin-6; SOD, superoxide dismutase; CAT, catalase; GPx, glutathione peroxidase.

At present, there is a growing amount of evidence that shows the neuroprotective role of antioxidants in gliosis (Table 1 and Figure 1). In recent studies, it has been proposed that lunasin has a bioactive action on obesity, since, in studies carried out in mice, it mediates the anti-inflammatory response and its mechanism of action helps to obtain a better response from type 1 helper T cells against low immune activity caused by obesity [117,118,119]. In the study of Sharma et al., they showed that treatment with hydroethanolic hull extract of *Juglans regia* L. reverted the neuronal degeneration, spongiosis and gliosis in cerebral cortex of rats in a model of oxidative damage induced with isoprenaline. Additionally, this extract increases catalase (CAT), superoxide dismutase (SOD), glutathione reductase (GR) activities, decreases the levels of malondialdehyde (MDA) and advanced oxidation protein product (AOPP) levels in brain [120]. Del Monte et al. showed the neuroprotective efficacy of the treatment with eye drops with nano-micellar formulations, containing melatonin/agomelatine in a rat model of hypertensive glaucoma. This prevented gliosis-related inflammation and recovered retinal dysfunction [121]. Anyanwu et al. showed the neuroprotective effect of *Costus afer* aqueous leaf extract against a low dose of heavy metal mixture (PbCl_2_, 20 mg/kg; CdCl_2_, 1.61 mg/kg; HgCl_2_, 0.40 mg/kg only)-induced neurotoxicity in rats. All extracts induced a protective effect against reactive gliosis and glial cell proliferation observed in the brain, diminished MDA levels and pro-inflammatory cytokine IL-6, and improved the SOD, CAT and glutathione (GSH) levels [122]. In an animal model of autism induced by LPS, it was shown that exenatide significantly reduced the hippocampal gliosis. Additionally, a positive effect was evident on brain serotonergic and GABAergic pathways exerted by exenatide [123]. In other studies, *Malva sylvestris* extract and fisetin (a phytomedicine-based potent antioxidant) showed a decrease in the astrogliosis and inflammatory or oxidative stress in damage induced by LPS in animal models of depression and memory impairment [124,125]. A recent study Koza at al. showed that protocatechuic acid (PCA), a phenolic metabolite of the parent anthocyanin, koumarin of some berries, diminishes gliosis in a transgenic mouse model of amyotrophic lateral sclerosis (ALS). The antioxidant reduced astrogliosis and microgliosis in spinal cord, protected spinal motor neurons from apoptosis, and maintained neuromuscular junction integrity in transgenic mice [126]. Other authors also showed the neuroprotective effects of antioxidant genistein and dichloroacetate, a metabolic modulator, the compounds alleviated gliosis in the spinal cord of SOD-1-G93A mice model of ALS through a decrease in the production of pro-inflammatory cytokines and modulation of mitochondria [127,128]. The administration of α-asarone and molecular hydrogen has also shown decreased reactive gliosis in a model of spinal cord injury through its antioxidant properties [129,130].

In the work of Hazzaa et al., the protective effect of *Allium sativum* powder was shown in a monosodium glutamate (MSG)-induced excitotoxicity in rats; the extract contained the antioxidants diallyl disulphide, carvone, diallyl trisulfide, and allyl tetrasulfide. The powder prevented MSG-induced neurotoxicity, and improved the short-term memory and gliosis by enhancing SOD activity and reducing the lipid peroxidation in the brain. It also reduced caspase-3 and increased ki-67 expression, causing retained brain tissue architecture [131]. Lee et al. also showed the protective effect of laminarin, a polysaccharide with antioxidant functions, against the damage induced by transient forebrain ischemia in gerbils. The authors showed that pretreatment with this compound protected neurons, attenuated reactive gliosis, and reduced pro-inflammatory M1 microglia in the CA1 field following ischemia [132]. The protective role of dimethyl fumarate or Korean red Ginseng, compounds with antioxidant and anti-inflammatory properties, against gliosis induced by hypoxic ischemia, has also been shown. The authors demonstrated that these compounds sustained neuroprotection in a Nrf-2-dependent manner using Nrf2^−/−^ mice [133]. The *Populus tomentiglandulosa* extract has also inhibited neuronal loss and alleviates gliosis observed in the CA1 hippocampal area in the transient global cerebral ischemia model in gerbils [134]. The administration of galantamine, a cholinesterase inhibitor, and *Glehnia littoralis* extract has also showed reduced astrogliosis and up-regulation of the activities of CAT and SOD-1, observed in neonatal hypoxia ischemia [135,136]. Hydroquinone, an immunoregulator compound with antioxidant properties, reduced the gliosis observed in a gerbil model of transient cerebral ischemia [137]. In the work of Wang et al., wogonin (5,7-dihydroxy-8-methoxy flavone) decreased the vacuolization and nuclear pyknosis in the neuronal cells and focal gliosis in brain of rats exposed to gamma irradiation. Additionally, this compound increases GSH, SOD, CAT and glutathione peroxidase (GPx), nuclear factor erythroid 2-related factor 2 (Nrf2) and heme oxygenase-1 (HO-1) mRNA and protein expression and diminishes MDA, TNF-α, IL-1β and IL-6 levels and NF-κB mRNA and protein expression [138]. In the same model, it has been shown that taurine, a sulfur-holding nonessential amino acid with antioxidants properties, ameliorated vacuolization and nuclear pyknosis in the neuronal cells and focal gliosis in cerebral cortex of rats, diminished MDA, NO, and TNF-α levels, as well as cytochrome-c, and increased caspases-9 and -3 along with GSH, SOD, CAT and GPx [139]. Additionally, Liu et al. showed that fruitless wolfberry-sprout extract (FWE) inhibited Aβ fibrillation, attenuated oxidative stress, decreased gliosis, and cytokines release in brain in an Alzheimer’s disease model of transgenic mice [140]. The work of Sadagurski et al. showed, in rats, that three compounds, acarbose (ACA), 17-α-estradiol (17αE2), and nordihydroguaiaretic acid (NDGA), reduced the hypothalamic inflammation and decreased hypothalamic reactive gliosis associated with aging in males [141]. Song et al. showed that the use of rosemary extract reduced astrocytosis in a model of traumatic brain injury. In this work, the authors showed that the extract prevented ROS generation, an increase in the protein levels of IL-1β, IL-6 and TNF-α in hippocampus and a decrease in SOD, GPx and CAT activity induced by the trauma [95]. It has been shown that thymoquinone (TQ), a phytochemical compound obtained from the plant *Nigella sativa*, attenuated astrogliosis and neurodegeneration in a model of temporal lobe epilepsy. TQ also decreased MDA levels, mossy fiber sprouting and seizure activity [142].

Melatonin, a N-acetyl-5-methoxytriptamine, is mainly synthesized by the pineal gland of all mammals and is involved in the regulation of multiple physiological processes, including circadian rhythm. This compound has been studied in vitro and shows hydroxyl scavenger capacities [143]. The direct antioxidant and free radical capacity of melatonin are mainly due to its electron-rich aromatic indole ring, which makes it a potent electron donor that reduces oxidative stress [144,145,146]. Tu et al. showed that melatonin inhibited the gliosis activation and pro-inflammatory cytokine production in Muller cells throughout the MEG3/miR-204/Sirt1 axis in an experimental model of diabetic retinopathy [147]. In another model of spinal cord injury, it was shown that melatonin attenuated gliosis and down-regulated the pro-inflammatory markers iNOS, IL-1β and TNF-α expressions [148]. The protective effect of melatonin against gliosis, neuroinflammation and neurodegeneration also has been shown in a BBB dysfunction rat model, in a chronic cerebral hypoperfusion mice model and in a scopolamine-induced amnesia mice model [149,150,151].

Curcumin is a phenolic compound, named diferuloylmethane; [1,7-bis-(4-hydroxy-3-methoxyphenyl)-1,6-heptadiene-3,5-dione] and is an extremely potent, lipid-soluble antioxidant. Structurally, curcumin consists of two ortho-methoxylated phenols and a beta-diketone moiety, and they are all conjugated. The free radical scavenging capacity can arise from the phenolic HO group or from the CH2 group of the β-diketone moiety. A free radical can undergo electron transfer from either of these two sites [152,153,154]. In relation to curcumin evaluated in human subjects, the effect of this antioxidant has been evidenced in atrophy, migraine, spinal cord injury, cognitive deficit, neurofibromatosis, Alzheimer Disease, and multiple sclerosis [155,156].

Quercetin is a polyphenolic compound that exhibits strong antioxidant activity. The compound is a flavonoid named 3,5,7,3′,4′-pentahydroxyflavone and is a basic structure consisting of three phenolic groups. Its reductor effect occurs when it donates a proton and becomes a radical itself, resulting in an unpaired electron delocalized by resonance, making the radical too low in energy to be reactive. It is known that the three structural groups in quercetin have the ability to remain as antioxidants when reacting with free radicals: the B ring o-dihydroxyl groups, the 4-oxo group in conjugation with the 2,3-alkene, and the 3- and 5-hydroxyl groups [157,158,159,160,161].

The three antioxidants displayed above have been studied in the clinical field, specifically in neurodegenerative diseases. Quercetin has been evaluated in clinical studies, where the effect of this antioxidant is assessed in neuropathies, Alzheimer Disease, optic neuritis, and tinnitus. In relation to melatonin, it has been evaluated in clinical assays related to cranial nerve diseases, multiple sclerosis, epilepsy, sleep disorders, spinal cord injuries, migraine, trauma brain injury, dementia, Huntington, Alzheimer and Parkinson Diseases [162,163,164,165,166,167,168,169,170,171,172,173,174,175,176,177,178,179,180,181,182,183,184,185,186,187,188,189,190,191,192,193,194]. Of note, some of these diseases have also been studied in experimental models, evaluating the neuroprotective effect of these compounds mainly by their antioxidant and anti-inflammatory properties [195,196,197,198,199,200,201,202,203,204,205]. Moreover, the effects of quercetin, melatonin and curcumin have been explored in obese patients in different studies, but they have not proposed gliosis as an important comorbidity [206,207,208,209,210,211,212,213,214]. Other antioxidants that can be proposed as possible neuroprotectors in gliosis are resveratrol [214,215,216], epigallocatechin-3-gallate [217], 4-hydroxy-tempo (TEMPOL) [218], astilbin [219], 2-hydrazino-4,6-dimethylpyrimidine [220], L-carnitine and exendin-4 [221,222], simvastatin [223], creatine [224], ellagic acid [225], cannabidiol [226], celastrol [227], ebselen [228], Idenebone [229] and N-acetylcysteine [230].

**Table 1:** Potential antioxidants for gliosis treatment in obese patients.

Antioxidant	Model	Effect
*Juglans regia* L. (hydroethanolic hull extract, 300 mg/kg oral gavage)	Isoprenaline (ISO) induced oxidative damage in brain of Wistar rats	Reverted the neuronal degeneration, spongiosis and gliosis in cerebral cortex, increased catalase (CAT), superoxide dismutase (SOD), glutathione reductase (GR) activities and decreased the levels of malondialdehyde (MDA) and advanced oxidation protein product (AOPP) in rats exposed to ISO. The extract also restored total antioxidant status (TAS) and total thiols (TTH) after ISO administration [120]
*Costus afer* (aqueous leaf extract, 750, 1500 and 2250 mg/kg body weight)	Wistar rats exposed to low-dose heavy-metal-mixture (lead, cadmium and mercury)	Brain protective effect against reactive gliosis and glia cell proliferation, diminished MDA levels and pro-inflammatory cytokine IL-6 and improved the levels of SOD, CAT and glutathione (GSH) in comparison with heavy-metal-mixture-treated rats [122]
Exenatide (a long-acting glucagon-like peptide 1, 20 µg/kg/day)	Autism model induced by the administration of lipopolysaccharide (LPS) to pregnant rats	Reduced the inflammation and hippocampal gliosis, and has a protective effect on brain serotonergic and GABAergic function (increasing 5-HIAA, GAD-67, and NGF levels). Positive effects on behavioral disorders. A decrease in TNF- α levels and an increase in SOD activity in brain [123]
*Malva sylvestris* (250 mg/kg) intragastrical per day for seven consecutive days	LPS-induced depression-like mice	Decreased the apoptosis and astrogliosis in the cortex and the CA1 region of hippocampus and alleviated anxiety. Decreased inflammatory markers (IL-1β/6 and TNF-α) and up-regulated IL-4 levels. [124]
*Fisetin* (20 mg/kg/day i.p. for 2 weeks, 1 week pre-treated to LPS and 1 week co-treated with LPS)	Oxidative damage induced by LPS in mice	Decreased oxidative stress and gliosis and activated phosphorylated c-JUN N-terminal Kinase (p-JNK) in the hippocampus. Additionally, decreased the inflammatory Toll-like Receptors (TLR4)/cluster of differentiation 14 (CD14)/phospho-nuclear factor kappa (NF-κB) signaling and attenuated other inflammatory mediators (tumor necrosis factor-α (TNF-α), interleukin-1 β (IL1-β), and cyclooxygenase (COX-2) [125]
*Allium sativum* (200 mg/kg of body weight)	Monosodium glutamate (MSG)-induced neurotoxicity in Wistar albino rats model	Improved short-term memory and gliosis by enhancing SOD activity and reducing the lipid peroxidation in the brain. It also reduced caspase-3 and increased ki-67 expression, causing retained brain tissue architecture [131]
Laminarin (50 and 100 mg/kg)	Brain injury induced by ischemic insult in gerbils	Pretreatment with this compound protected neurons, attenuated reactive gliosis, and reduced pro-inflammatory M1 microglia in the CA1 field following ischemia [132]
*Populus tomentiglandulosa* (Ethanol extract 200 mg/kg)	Brain injury induced by ischemic insult in gerbils	Inhibited neuronal loss and alleviated gliosis observed in CA1 hippocampal area [134]
*Glehnia littoralis* (Ethanol extract, 100 and 200 mg/kg)	Brain injury induced by ischemic insult in gerbils	Reduced astrogliosis and up-regulated SOD-1 expression in CA1 hippocampal area [135]
Wogonin (a flavone, 30 mg/kg)	Neurotoxicity induced by γ-radiation in rats.	Decreased the vacuolization and nuclear pyknosis in the neuronal cells and focal gliosis in cerebral cortex. Additionally, this compound increased GSH, SOD, CAT and GPx, nuclear factor erythroid 2-related factor 2 (Nrf2) and heme oxygenase-1 (HO-1) mRNA and protein expression and diminished MDA, TNF-α, IL-1β and IL-6 levels and NF-κB mRNA and protein expression [138]
Epigallocatechin-3-gallate (10 mg/kg body weight/day intragastrical)	Acrylamide-induced apoptosis in Sprague-Dawley rats	Inhibited oxidative stress by enhancing the activity of antioxidant enzymes and glutathione levels and reducing the formation of reactive oxygen species and lipid peroxidation in the cerebral cortex. Inhibited DNA damage and apoptosis [231]
Melatonin (10 mg/kg/day)	C57BL/6 mice model of diabetic retinophaty Acute spinal cord injury model in rats	Inhibited the gliosis activation and pro-inflammatory cytokine production (VEGF, TNF-α, IL1-β and IL-6) throughout the MEG3/miR-204/Sirt1 axis. In the spinal cord injury model, melatonin attenuated gliosis and down-regulated the pro-inflammatory markers iNOS, IL-1β and TNF-α expressions. Moreover, it increased SOD, CAT and GPx levels, decreased MDA content, down-regulated caspase-3, Bax and GFAP expressions and up-regulated Bcl-2 expression [147,148]
Acarbose (1000 ppm), 17-α-estradiol (14.4 ppm), and nordihydroguaiaretic acid (2500 ppm) mixed in the diet	Male mouse model of aging	Reduced the hypothalamic inflammation (TNF-α and GFAP) and decreased hypothalamic reactive gliosis associated with aging in males [141].
Quercetin (30 mg/kg/day, intraperitoneal in LPS model and 10 mg/kg, intraperitoneal in trauma brain injury)	Administration of LPS in mice Repetitive traumatic brain injury model in mice	In LPS model, reduced activated gliosis and prevented neuroinflammation in the cortex and hippocampus. Rescued the mitochondrial apoptotic pathway and neuronal degeneration by regulating Bax/Bcl2, and decreased activated cytochrome c, caspase-3 activity and cleaving PARP-1 in the cortical and hippocampal regions. In trauma brain injury, reduced gliosis in CA1 [160,161]
Curcumin (200 mg/kg by oral gavage in nanoparticles model and 1 μM and 50 μM in astrocyte cell lines)	Neurotoxicity induced by zinc oxide nanoparticles in rats Oxidative stress induced by hydrogen peroxide in A172 (human glioblastoma cell line) and HA-sp (human astrocytes cell line derived from the spinal cord) astrocytes	In nanoparticle models, ameliorates the deleterious effect of neurotoxins on the cerebellar cortex through its antioxidant (GPx and total antioxidant capacity increase and nitric oxide and MDA decrease), antiapoptotic (caspase-3 and p53 decrease), and anti-inflammatory (COX-2, IL-6 and TNF-α decrease) effects [155] In the astrocytes cell lines, protects astrocytes from hydrogen peroxide-induced oxidative stress but also reverses the inflammation, apoptosis and mitochondria fragmentation and dysfunction induced by oxidative stress (inhibiting up-regulation of GFAP, vimentin and Prdx6) [156]

## 11. Conclusions

Obesity is one of the main public health problems worldwide, in addition to being associated with comorbidities such as type 2 diabetes and other metabolic diseases that cause alterations in several regions of the body. Neuroinflammation in obese patients is clearly evident; nevertheless, gliosis is not considered an important comorbidity. This disease has not been appropriately treated, diagnosed and prevented; therefore, it is a risk factor for the appearance of neurodegenerative diseases. The literature that were consulted mainly focused on the damage that obesity causes to the hypothalamus, and the structure associated with the ingestion and satiety processes; nevertheless, damage due to neuroinflammation has also been described in frontal cortex, parietal cortex, basal ganglia, thalamus, hippocampus and cerebellum. In this review, we point out the most important aspects of gliosis in obese patients, the available diagnosis tools and antioxidants’ capacity for the prevention and treatment of this condition.

Antioxidants are efficient in reducing neuroinflammation and promoting neuroprotection in models of nervous system damage; however, studies evaluating the effect of antioxidants in gliosis in obese patients are limited. According to what we found in this review, we can mention that curcumin, quercetin and melatonin could be the most promising antioxidants for the management of gliosis in patients with obesity, due to their mechanism of action and their safety. In addition, further research is warranted regarding Acarbose, 17-α-estradiol and nordihydroguaiaretic acid, because they have been shown to reduce neuroinflammation specifically in the hypothalamus, the brain region most affected by obesity. This was conducted with the aim of proposing novel therapeutic targets in gliosis due to obesity. However, most of these studies have been reported in animal models; therefore, further clinical studies in human are urgently needed.
